# Timeliness of Yellow Fever Surveillance, Central African Republic

**DOI:** 10.3201/eid2006.130671

**Published:** 2014-06

**Authors:** Antoine Rachas, Emmanuel Nakouné, Julie Bouscaillou, Juliette Paireau, Benjamin Selekon, Dominique Senekian, Arnaud Fontanet, Mirdad Kazanji

**Affiliations:** Institut Pasteur, Bangui, Central African Republic (A. Rachas, E. Nakouné, J. Bouscaillou, B. Selekon, M. Kazanji);; Institut Pasteur, Paris, France (A. Rachas, J. Bouscaillou, J. Paireau, A, Fontanet);; Ministry of Health, Bangui, (D. Senekian);; Conservatoire National des Arts et Métiers, Paris (A. Fontanet)

**Keywords:** yellow fever, viruses, epidemiology, public health, surveillance, timeliness, Central African Republic

## Abstract

During January 2007–July 2012, a total of 3,220 suspected yellow fever cases were reported in the Central African Republic; 55 were confirmed and 11 case-patients died. Mean delay between onset of jaundice and case confirmation was 16.6 days. Delay between disease onset and blood collection could be reduced by increasing awareness of the population.

Because the number of reported cases of yellow fever has increased over the past 2 decades, it is considered a reemerging disease (*1*,*2*). The World Health Organization (WHO) requires that all affected countries report yellow fever cases. Increasing risk for resurgence, potential severity of an epidemic, and possibility of preventing its spread by vaccination make early detection of yellow fever outbreaks essential, especially in a country such as the Central African Republic, where access to healthcare is difficult because of security concerns in several areas. 

There are minimal data for yellow fever surveillance in Africa. The purpose of this study was to describe the timeliness, which was defined as the delay between date of onset of jaundice reported by the patient and date of an ELISA result, of the yellow fever surveillance system in the Central African Republic and identify temporal and spatial patterns and factors associated with delays in reporting.

## The Study

This study was conducted as part of epidemiologic surveillance activities of the Ministry of Public Health of the Central African Republic. Data were obtained through the yellow fever surveillance system and approved by the Ministry of Health and WHO. All suspected cases reported during January 2007–July 2012 were included. A suspected case of yellow fever was defined as an acute onset of fever in a patient followed by jaundice within 2 weeks (*3*).

All blood samples from patients with suspected cases were tested at the Institut Pasteur (Bangui, Central African Republic) (IPB) for yellow fever virus–specific IgM by using the ELISA developed by the US Centers for Disease Control and Prevention (*4*). Positive samples were sent to the regional reference laboratory at the Institut Pasteur in Dakar, Senegal, where a plaque-reduction neutralization test (PRNT) was performed for case confirmation. A suspected yellow fever case was ruled out if ELISA or PRNT results were negative. When a suspected case was not ruled out by ELISA, health authorities were informed and an investigation was conducted so that vaccination could be implemented without delay if the case was confirmed.

The main study outcome was timeliness of the yellow fever surveillance system. Each intermediate step was studied: collection (from onset of jaundice to blood sample collection), field storage (from sample collection to shipping), transportation (from shipping to reception at IPB) and testing (from reception of sample to ELISA result).

Survival analysis was performed to determine how the following factors affected time to confirmed cases: age, sex, onset during the rainy season (May 1–October 31), province of residence, history of vaccination against yellow fever, and year of onset. Because the proportional hazard assumption was not verified for several variables, we used a parametric survival model and assumed a log-normal distribution of event times to estimate mutually adjusted time ratios. Subgroups were analyzed by confirmation status (confirmed or ruled out cases). Data were analyzed with Stata software version 11.0 (StataCorp LP, College Station, TX, USA) Maps were drawn with ArcGIS version 10.1 (Esri, Redlands, CA, USA).

During January 2007–July 2012, a total of 3,220 suspected cases of yellow fever were reported to IPB (Technical Appendix Figure 1). Suspected cases were reported in all provinces but mostly in Bangui (32.7%) and in neighboring Ombella M’Poko Province (24.7%) (Technical Appendix Figure 2). Median age of patients with suspected cases was 21 years (interquartile range 10–30 years) and 57.5% were men. Only 21.9% reported having been vaccinated against yellow fever virus within the previous decade (Table 1).

**Table 1:** Characteristics of 3,220 case-patients with suspected yellow fever, Central African Republic, 2007–2012*

Characteristic	Suspected case-patients, n = 3,220	Confirmed case-patients, n = 55	Ruled out case-patients, n = 3,165	p value
Age, y				
<14	1,017 (31.7)	9 (16.4)	1,008 (32.0)	NA
15–24	882 (27.5)	22 (40.0)	860 (27.3)	NA
25–34	673 (21.0)	16 (29.1)	657 (20.9)	NA
≥35	633 (19.8)	8 (14.6)	625 (19.8)	0.020
Median (IQR)	21 (10–30)	23 (17–28)	21 (10–31)	0.32
Sex				
M	1,851 (57.5)	35 (63.6)	1,816 (57.4)	NA
F	1,366 (42.5)	20 (36.4)	1,346 (42.6)	0.36
Onset during rainy season				
No	1,446 (45.4)	27 (50.0)	1,419 (45.3)	NA
Yes	1,738 (54.6)	27 (50.0)	1,711 (54.7)	0.50
Province of residence				
Bangui	1,053 (32.7)	11 (20.0)	1,042 (32.9)	NA
Ombella M’Poko	794 (24.7)	30 (54.5)	764 (24.1)	NA
Lobaye	189 (5.9)	5 (9.1)	184 (5.8)	NA
Sangha Mbaéré	20 (0.6)	0	20 (0.6)	NA
Mambéré Kadéi	43 (1.3)	0	43 (1.4)	NA
Nana Mambéré	40 (1.2)	0	40 (1.3)	NA
Ouham Péndé	178 (5.5)	1 (1.8)	177 (5.6)	NA
Ouham	111 (3.5)	0	111 (3.5)	NA
Nana Grigbizi	71 (2.2)	0	71 (2.2)	NA
Kemo	88 (2.7)	0	88 (2.8)	NA
Bamingui Bangoran	23 (0.7)	0	23 (0.7)	NA
Ouaka	188 (5.8)	1 (1.8)	187 (5.9)	NA
Basse Kotto	178 (5.3)	4 (7.3)	174 (5.5)	NA
Vakaga	33 (1.0)	0	33 (1.0)	NA
Haute Kotto	114 (3.5)	3 (5.5)	111 (3.5)	NA
Mbomou	78 (2.4)	0	78 (2.5)	NA
Haut Mbomou	19 (0.6)	0	19 (0.6)	NC
Vaccination against yellow fever				
Never	2,301 (71.5)	28 (50.9)	2,273 (71.8)	NA
>10 y ago	160 (5.0)	3 (5.5)	157 (5.0)	NA
≤10 y ago	705 (21.9)	11 (20.0)	694 (21.9)	NA
Unknown date	54 (1.7)	13 (23.6)	41 (1.3)	<0.001

*Values are no. (%) except as indicated. NA, not applicable; IQR, interquartile range; NC, not computable.

**Figure 1:**
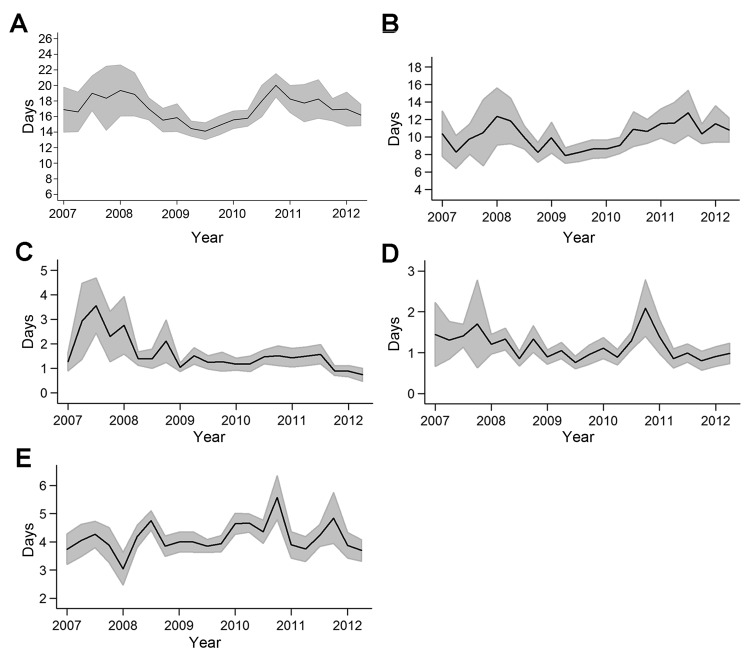
Temporal pattern of mean time (delay between date of onset of jaundice reported by the patient and date of an ELISA result) for A) yellow fever surveillance, B) blood sample collection, C) field storage of samples, D) transportation of samples, and E) testing of samples, Central African Republic, 2007–2012. Shaded areas indicate 95% CIs.

Mean time to confirmation was 16.6 days (95% CI 16.2–16.9 days). Mean delay was 9.9 days (95% CI 9.5–10.2 days) for blood sample collection, 1.5 days (95% CI 1.4–1.6 days) for field storage, 1.1 days (95% CI 1.0–1.1 days) for transportation, and 4.2 days (95% CI 4.1–4.3 days) for testing. Detection of yellow fever (Figure 1, panel A) and delay for blood collection (Figure 1, panels B–E) were shortest in 2009.

**Figure 2:**
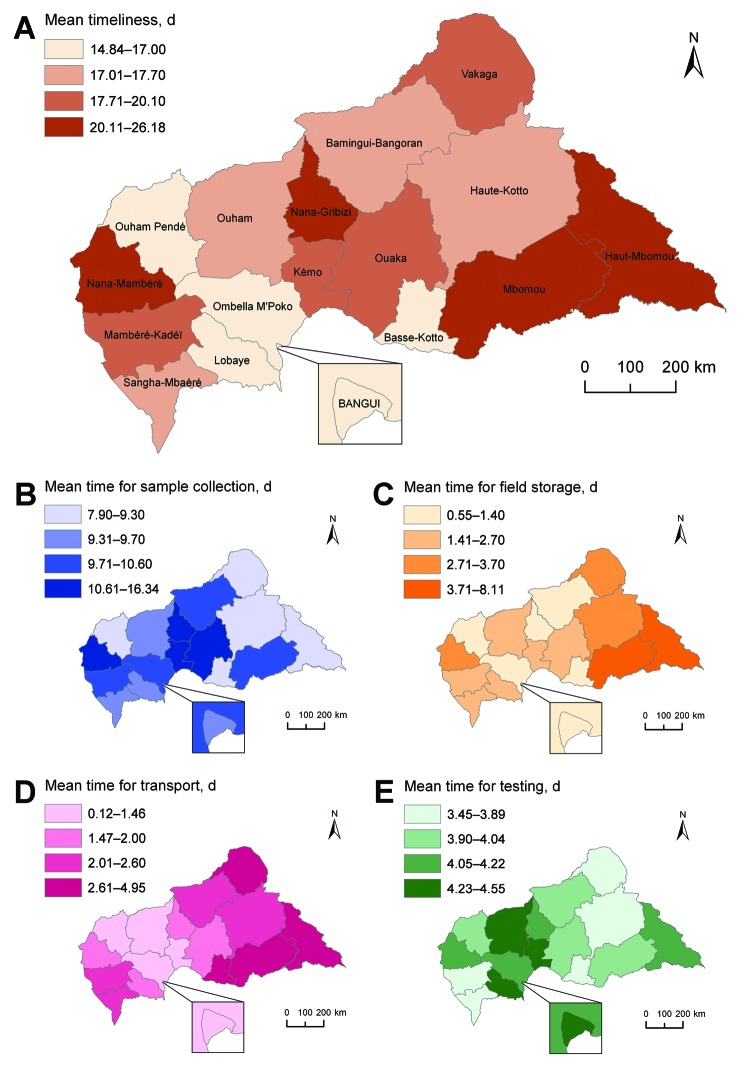
Spatial pattern of mean time (delay between date of onset of jaundice reported by the patient and date of an ELISA result for A) yellow fever surveillance, B) mean time for blood sample collection, C) mean time for field storage of samples, D) mean time for transportation of samples, and E) mean time for testing of samples, by province, Central African Republic, 2007–2012.

Mean time until blood collection was shortest in Bangui (14.8 days) and surrounding areas and longest in Mbomou (26.2 days) (Figure 2, panel A). Mean delay for blood sample collection was shorter in eastern and northern regions of the country and longer in central and western regions (Figure 2, panel B). Mean length of transportation varied along a west–east gradient and ranged from 0.1 days in Bangui to 4.9 days in Haut Mbomou (Figure 2, panel D). A longer period to blood sample testing for the eastern part of the country was related to longer times of field storage and transportation (Figure 2, panels C, D). These areas are least accessible because of distance and security concerns. Transportation time was longer in the southwestern region despite its relative proximity to Bangui. Mean time for testing remained stable at ≈4–5 days (Figure 2, panel E).

Province of residence and year of onset were associated with a shorter period to diagnosis (p<0.001) (Table 2). Time to blood sample testing increased with patient age (p<0.001). No association was found between other characteristics and time required to confirm cases.

**Table 2:** Factors associated with timeliness of yellow fever surveillance, Central African Republic, 2007–2012*

Factor	Mean timeliness, d	Adjusted time ratio (95% CI)	p value†
Age, y			
<14	15.3	1.00	NA
15–24	16.5	1.13 (1.08–1.18)	NA
25–34	16.8	1.15 (1.09–1.21)	NA
≥35	18.6	1.21 (1.15–1.27)	<0.001
Sex			
M	16.6	1.00	NA
F	16.5	1.01 (0.98–1.05)	0.43
Onset during rainy season			
No	16.7	1.00	NA
Yes	16.5	1.01 (0.97–1.04)	NA
Province of residence			
Bangui	14.8	1.00	NA
Ombella M’Poko	15.7	1.07 (1.02–1.12)	NA
Lobaye	16.8	1.18 (1.09–1.28)	NA
Sangha Mbaéré	17.6	1.17 (0.93–1.46)	NA
Mambéré Kadéi	18.0	1.21 (1.03–1.41)	NA
Nana Mambéré	20.7	1.43 (1.22–1.67)	NA
Ouham Péndé	15.5	1.10 (1.01–1.19)	NA
Ouham	17.0	1.19 (1.08–1.32)	NA
Nana Grigbizi	22.9	1.56 (1.39–1.77)	NA
Kemo	19.4	1.35 (1.21–1.51)	NA
Bamingui Bangoran	17.5	1.24 (1.01–1.53)	NA
Ouaka	19.2	1.34 (1.24–1.46)	NA
Basse Kotto	16.5	1.19 (1.10–1.29)	NA
Vakaga	20.1	1.38 (1.16–1.65)	NA
Haute Kotto	17.4	1.25 (1.13–1.39)	NA
Mbomou	26.2	1.86 (1.66–2.09)	NA
Haut Mbomou	23.9	1.72 (1.37–2.15)	<0.001
Vaccination against yellow fever			
Never	16.6	1.00	NA
>10 y ago	16.9	1.01 (0.93–1.09)	NA
≤10 y ago	16.3	1.01 (0.96–1.05)	NA
Unknown date	16.1	1.06 (0.92–1.23)	0.85
Year of onset			
2007	17.8	1.00	NA
2008	17.3	1.02 (0.95–1.09)	NA
2009	14.7	0.91 (0.85–0.98)	NA
2010	16.8	1.04 (0.97–1.12)	NA
2011	17.8	1.10 (1.02–1.18)	NA
2012	16.6	1.05 (0.96–1.15)	<0.001

*Adjusted time ratios are from a parametric survival model assuming log-normal distribution of the event times. A time ratio represents a relative increase in time between 2 groups. Timeliness was defined as the delay between the date of onset of jaundice (reported by the patient) and the date of ELISA result. NA, not applicable. †By global Wald test.

A total of 55 yellow fever cases were confirmed and 11 case-patients died. Of the confirmed case-patients, 22 (40.0%) were 15–24 years of age, 35 (63.6%) were male, and for 13 (23.6%) date of vaccination against yellow fever virus was unknown. Age and history of vaccination differed between patients who had confirmed yellow fever cases and those who did not (Table 1). Timelines were similar for confirmed and ruled out cases.

## Conclusions

This study shows that in the Central African Republic, a country with limited health care and transportation facilities, confirmation of a yellow fever case takes 2–3 weeks. Approximately 10 days (60% of the delay) are required from the onset of symptoms to blood collection.

A fast time to case confirmation was observed in 2009, which was largely caused by a decrease in time required for blood collection, could be related to greater public awareness resulting from a yellow fever outbreak in Ombella M’Poko and Lobaye (*5*) and from a large outbreak of hepatitis E. This study showed an unexpected association between younger age and more rapid time to diagnosis, which was caused mainly by a shorter delay until blood collection, suggesting that parents have their children tested earlier. These 2 results indicate that the delay to blood collection could be reduced by better awareness of the population to the need for testing when they have symptoms compatible with yellow fever.

The 4–5 days of delay until testing is below the threshold of 7 days recommended by WHO. However, it could be decreased by implementation of a method of yellow fever detection (Bioplex Technology; BioRad Laboratories, Hercules, CA, USA), which is now in progress at IPB.

The need for confirmation by PRNT might delay vaccination because of time necessary for transportation of samples to Dakar and testing. The Central African Republic is landlocked and surrounded by countries to which yellow fever is endemic (*6*). Thus, PRNT for this region could be implemented at IPB, where biosafety level 3 facilities are functional. This factor could improve timeliness of diagnosis for rapid introduction of prevention measures.

Delays in reporting cases observed in this study are consistent with time between onset of symptoms of yellow fever in index case-persons and in persons infected by them, which is estimated to be ≈2 weeks (*7*). However, reasonable time for reporting is difficult to define. A comparison of delays found in this study with those in other countries would be helpful.

Technical Appendix Incidence rates of yellow fever cases, Central African Republic, 2007–2012.
