# Supplementary material for: Timeliness of Yellow Fever Surveillance, Central African Republic
Source: Emerg Infect Dis. 2014 Jun;20(6):1004–8. doi: 10.3201/eid2006.130671 (PMC4036780; doi:10.3201/eid2006.130671)
Supplement: Technical Appendix — Incidence rates of yellow fever cases, Central African Republic, 2007–2012. [file 13-0671-Techapp-s1.pdf]

Article DOI: http://dx.doi.org/10.3201/eid/2006.130671

Yellow Fever Surveillance, Central African Republic

Technical Appendix

Incidence rates of yellow fever cases, Central African Republic, 2007–2012.

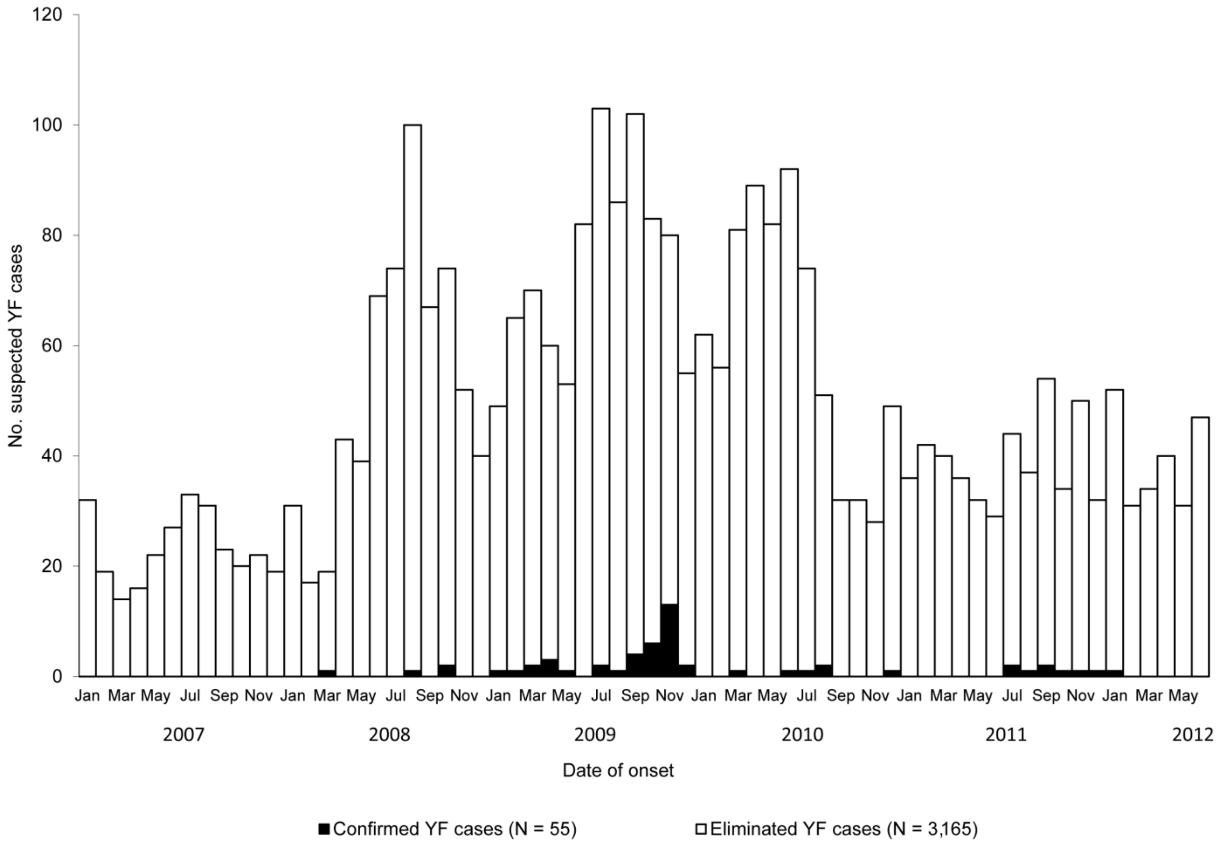

Technical Appendix Figure 1. Monthly incidence of yellow fever (YF) cases, Central African Republic, 2007–2012. Date of onset corresponds to onset of jaundice.

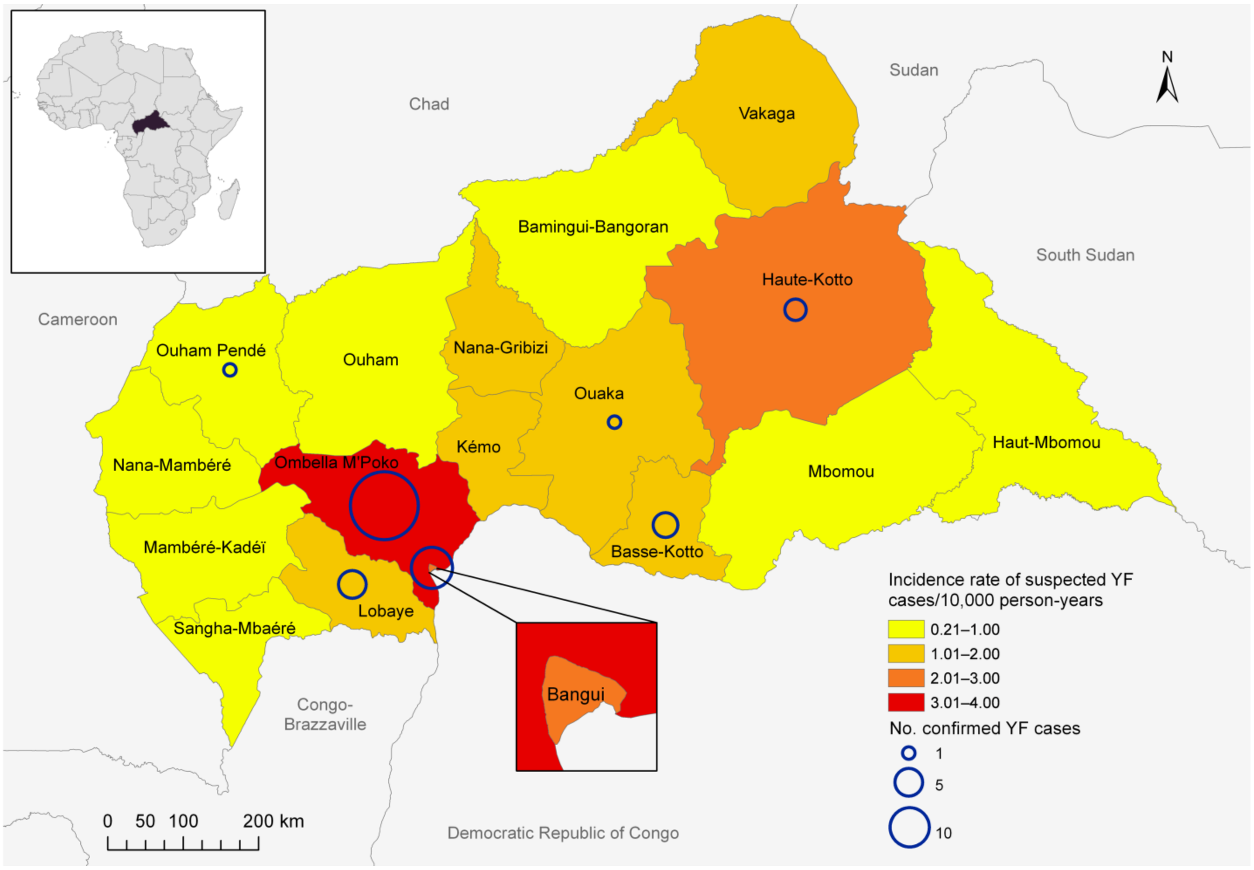

Technical Appendix Figure 2. Incidence rates of suspected cases of yellow fever (YF) and number of confirmed cases by province, Central African Republic, 2007–2012. Incidence rates were calculated on the basis of the last population estimate (2003 census).
